# Changes in flexibility but not in compactness underlie the thermal adaptation of prokaryotic adenylate kinases

**DOI:** 10.1093/evlett/qraf026

**Published:** 2025-08-06

**Authors:** Dimitrios - Georgios Kontopoulos, Ilias Patmanidis, Timothy G Barraclough, Samraat Pawar

**Affiliations:** Department of Life Sciences, Imperial College London, Silwood Park, Ascot, Berkshire, United Kingdom; LOEWE Centre for Translational Biodiversity Genomics, Frankfurt am Main, Germany; Senckenberg Research Institute, Frankfurt am Main, Germany; Institute of Physics, Carl von Ossietzky Universität Oldenburg, Oldenburg, Germany; Chemical and Pharmaceutical Biology, Groningen Research Institute of Pharmacy, University of Groningen, Groningen, the Netherlands; Department of Life Sciences, Imperial College London, Silwood Park, Ascot, Berkshire, United Kingdom; Department of Biology, University of Oxford, Oxford, United Kingdom; Department of Life Sciences, Imperial College London, Silwood Park, Ascot, Berkshire, United Kingdom

**Keywords:** thermal adaptation, microbes, adenylate kinases, enzyme structures

## Abstract

Understanding the structural changes that enable enzymes to remain active in extreme thermal conditions is of broad scientific interest for both fundamental and applied biological research. Three key mechanisms that underlie the thermal adaptation of enzymes are modifications in structural flexibility, compactness, and the contacts formed among amino acids. However, most previous studies on these topics have been limited to small sample sizes or a narrow taxonomic focus, and the importance of these factors to thermal adaptation remains poorly understood. In this study, we combined molecular dynamics simulations and phylogenetic comparative analyses to thoroughly analyze the structural factors underlying thermal adaptation in adenylate kinase—a key enzyme involved in cellular energy balance and homeostasis—across 70 prokaryotic species. We detect systematic increases in the flexibility of the enzyme with temperature, both across and within species. In contrast, structural compactness appears to be almost completely independent of temperature. Finally, we uncover a remarkable diversity in the number and types of amino acid contacts observed in different adenylate kinases that cannot be explained solely by temperature. Our results suggest that there are multiple paths toward the adaptation of prokaryotic adenylate kinases to extreme thermal environments and that these paths are generally accessible through changes in flexibility.

## Introduction

Changes in environmental temperature directly or indirectly affect most biological processes, from the activity and stability of enzymes to the functioning of entire ecosystems (Clarke, 2017; Knapp & Huang, 2022; Pawar et al., 2015; Vázquez et al., 2017). Understanding the mechanisms through which biological systems respond to temperature allows us not only to predict their future dynamics in the face of climate change but also to identify ways to modify them for ecological, environmental, or biotechnological purposes.

At the level of individual enzymes, both optimal temperature (where activity is maximized) and melting temperature (where half of the enzyme population is in the unfolded state) have been shown to correlate strongly and positively with growth temperature in microbes (Engqvist, 2018; Stark et al., 2022). This implies that the thermal adaptation of microbial growth rate should depend on concordant changes in the stability and activity of key enzymes, achieved through modifications of their three-dimensional structures (Feller, 2010).

**Figure 1.**
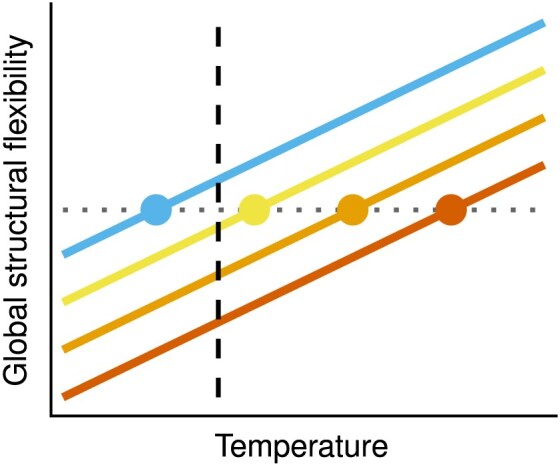
The relationship between the global structural flexibility of a given enzyme and temperature, according to the corresponding states hypothesis. The four colors represent orthologs of the same enzyme from different thermal environments. The corresponding states hypothesis posits that if flexibility is compared at the native temperature of each enzyme (circles), there should be no variation among orthologs. In other words, the interspecific slope (horizontal dotted line) should be zero. In contrast, if the four orthologs are compared at a common temperature (dashed vertical line), the coldest-adapted ortholog (blue) should be the most flexible.

The most commonly discussed hypothesis for the adaptation of enzyme structures to different thermal environments is the “corresponding states” hypothesis, introduced by Somero (1978). This hypothesis posits that orthologous enzymes from different thermal environments should have similar kinetic and thermodynamic properties at their respective native temperatures. One such property is global structural flexibility, which, for a given enzyme, should increase linearly with temperature due to the increase in kinetic energy. Because of this, the corresponding states hypothesis necessarily implies that, at a common (normalization) temperature, cold-adapted enzyme orthologs should be more flexible than warm-adapted orthologs (Figure 1).

A second, complementary hypothesis for the structural changes underlying the thermal adaptation of enzymes is an increase in structural compactness in high-temperature environments (Robinson-Rechavi et al., 2006; Tompa et al., 2016; Zhu et al., 1998). As enzyme structure becomes more compact, especially at its surface, its exposure to high-temperature solvents is reduced, decreasing the probability of denaturation. High structural compactness may also facilitate the formation of contacts among amino acid side chains—such as salt bridges—which can further stabilize the structure, making the denatured state less energetically favorable (Tompa et al., 2016).

Previous studies have found mixed support for the aforementioned hypotheses (e.g., Cipolla et al., 2012; Collins et al., 2003; Diessner et al., 2024; Fitter et al., 2001; Hajdú et al., 2008; Jenney et al., 2024; Karshikoff & Ladenstein, 1998; Maffucci et al., 2020; Nguyen et al., 2017; Szilágyi & Závodszky, 2000), but such studies generally suffer from certain common limitations. In particular, due to experimental and/or computational constraints, most studies tend to compare only a small number of orthologs—often as low as two—which are usually separated by long phylogenetic distances. This makes it difficult to distinguish between differences among orthologs that arose through thermal adaptation and those that arose randomly over millions of years of evolution (see Uyeda et al., 2018). Due to small sample sizes, few studies report quantitative estimates (i.e., intercepts and slopes) of the impacts of temperature on key structural variables across and/or within enzymes from different species. Such estimates could be directly integrated into data syntheses, allowing for systematic comparisons of the effects of temperature across different types of enzymes. On the other hand, studies with larger sample sizes often suffer from the opposite problem: They tend to focus on relatively narrow taxonomic groups, and therefore, it is often not possible to evaluate the generality of the conclusions drawn.

To thoroughly examine the structural changes involved in thermal adaptation of enzymes across a sufficiently wide taxonomic range, here we study the essential enzyme adenylate kinase (ADK). ADK catalyzes the reversible conversion of ATP and AMP to ADP. Balancing the levels of these three nucleotides is crucial for cellular homeostasis and metabolic versatility, and therefore, the ADK activity strongly determines organismal fitness (Couñago & Shamoo, 2005; Couñago et al., 2006, 2008; Peña et al., 2010; Tükenmez et al., 2016).

**Figure 2.**
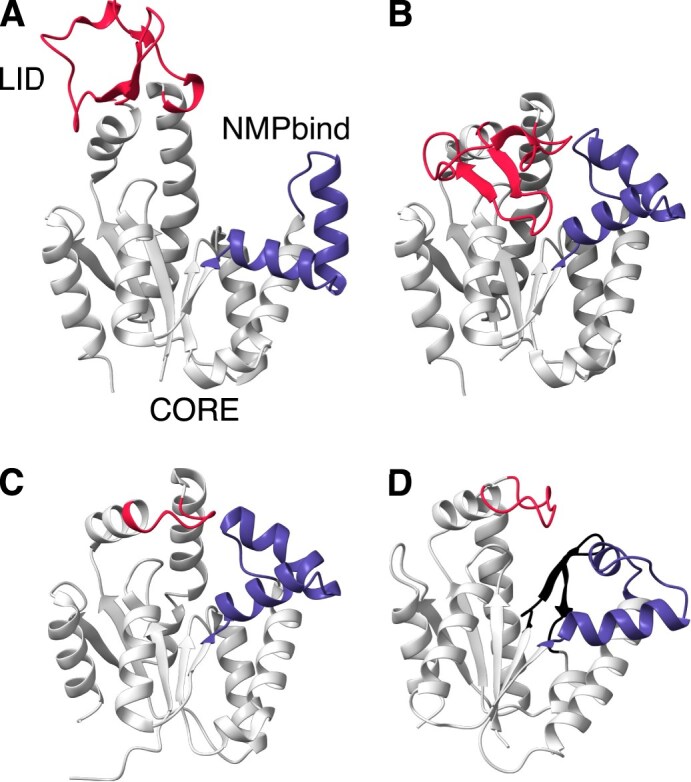
Main structural characteristics of prokaryotic adenylate kinases (ADKs). (A and B) Open and closed conformations of the ADK of the bacterium *Streptococcus pneumoniae* (PDB accession codes: 4NTZ and 4NU0). This ADK is monomeric and features a long LID domain (in red), in contrast to the monomeric ADK of the bacterium *Mycobacterium tuberculosis* (C; PDB accession code: 2CDN), which has a short LID domain. (D) The monomer of the trimeric ADK of the archaeon *Sulfolobus acidocaldarius* (PDB accession code: 1NKS). This ADK has a short LID domain and an additional beta hairpin (in black). The figure was generated with UCSF ChimeraX (v. 1.7.1; Pettersen et al., 2021).

ADKs have three major structural domains: (i) the CORE domain, which comprises the bulk of the structure, (ii) the NMPbind domain where AMP is bound, and (iii) the LID domain where ATP is bound (Li et al., 2015; Figure 2). The CORE domain is rigid, whereas the two other domains are flexible, allowing the structure to shift between catalytically inactive (open; e.g., Figure 2A) and active (closed; Figure 2B) conformations (Kovermann et al., 2017). Across prokaryotes, there are two main sources of variation in the ADK structure. First, instead of a long LID domain (Figure 2A and  B), some prokaryotic ADKs (e.g., that of *Mycobacterium tuberculosis*; Figure 2C) have a short LID (Bellinzoni et al., 2006). Second, while most prokaryotic ADKs are monomeric, some archaeal ADKs (e.g., that of *Sulfolobus acidocaldarius*; Figure 2D) have an additional beta hairpin, which enables them to operate in homotrimeric rather than in monomeric form (Vonrhein et al., 1998).

Using ADK as our study system, here we ask three main questions:

1. Do ADKs from different thermal environments occupy distinct regions of the structural parameter space?
2. Does environmental temperature systematically influence structural flexibility and compactness at the intra- and/or interspecific levels?
3. Does adaptation of ADKs to extreme thermal environments require the formation (or absence) of several specific amino acid contacts?

To address these questions, we manually curated a dataset of 70 ADK structures from diverse bacterial and archaeal species (with 17 phyla being represented) and thermal environments (Table S1). We first performed a phylogenetic analysis to evaluate the extent of amino acid sequence conservation among ADK orthologs. Next, we conducted molecular dynamics simulations to obtain estimates of flexibility, compactness, and the presence of amino acid contacts at various temperatures. Finally, we quantified the relationships between these factors and temperature through phylogenetic comparative methods.

## Results and discussion

### Dataset of prokaryotic ADKs and phylogenetic distribution

We classified ADKs in our dataset based on the thermal preferences of their corresponding species as (a) psychrophilic (optimal growth temperature up to 20 °C), (b) mesophilic (optimal growth temperature between 20 °C and 40 °C), (c) thermophilic (optimal growth temperature between 40 °C and 80 °C), or (d) hyperthermophilic (optimal growth temperature higher than 80 °C). Our dataset includes ADKs from 8 psychrophiles, 38 mesophiles, 16 thermophiles, and 8 hyperthermophiles. The LID domain is long in 49 and short in 21 ADKs, whereas 12 out of 70 ADKs possess a beta hairpin that enables them to form homotrimers.

**Figure 3.**
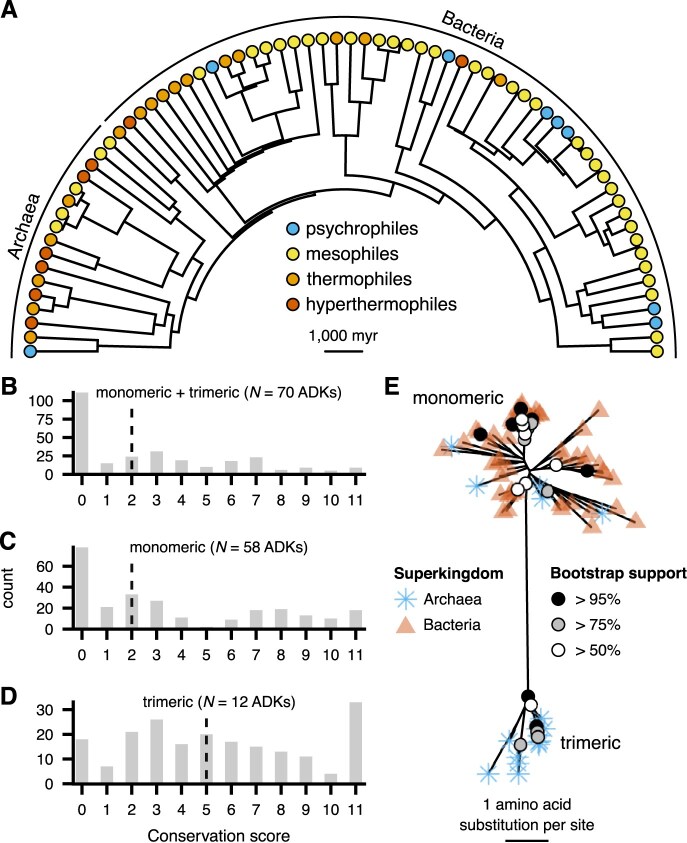
Overview of the 70 prokaryotic adenylate kinases (ADKs) included in this study. (A) Distribution of the four thermal groups across the species’ phylogeny, visualized using the phytools R package (v. 2.1-1; Revell, 2024). (B–D) Conservation scores for each column of the alignments of ADK amino acid sequences, obtained with Jalview (v. 2.11.3.3; Waterhouse et al., 2009). A value of 11 indicates columns where all sequences share a common amino acid, whereas lower values correspond to lower degrees of physicochemical conservation (Livingstone & Barton, 1993). The dashed vertical line in each panel stands for the median value. (E) The maximum-likelihood gene tree of all 70 ADK sequences, as estimated with IQ-TREE (v. 2.3.5; Minh et al., 2020) and visualized with the ggtree R package (v. 3.12.0; Yu et al., 2017). Branches with bootstrap support values greater than 50% are explicitly annotated.

Mapping these thermal groups on the species’ phylogeny (see the *Methods* section) revealed a scattered phylogenetic distribution (Figure 3A), which should provide adequate statistical power for addressing our three main questions. In particular, the phylogeny includes eight pairs of sister species (species immediately descending from a common node) that belong to different thermal groups. Neither the ADK type (monomeric or trimeric) nor the length of the LID is monophyletically distributed, possibly due to interspecific recombination events, similar to those previously reported for ADKs from the bacterial genus *Neisseria* (Feil et al., 1995, 1996).

### Amino acid sequence conservation

We next evaluated the degree of amino acid sequence conservation in each column of the alignment of (a) all ADKs, (b) monomeric ADKs, and (c) trimeric ADKs (Figure 3B–D). We found evidence for low conservation among most alignment columns, but especially for monomeric ADKs (Figure 3C). The sequences of trimeric ADKs appeared to be relatively more conserved (Figure 3D), which may be due to the need to maintain the trimerization interface. A gene tree reconstructed from the alignment of all 70 ADK amino acid sequences revealed two major clusters, one for monomeric and one for trimeric ADKs (Figure 3E). Most branches of the gene tree had very low bootstrap support, which is in line with the fact that most alignment columns were weakly conserved.

**Figure 4.**
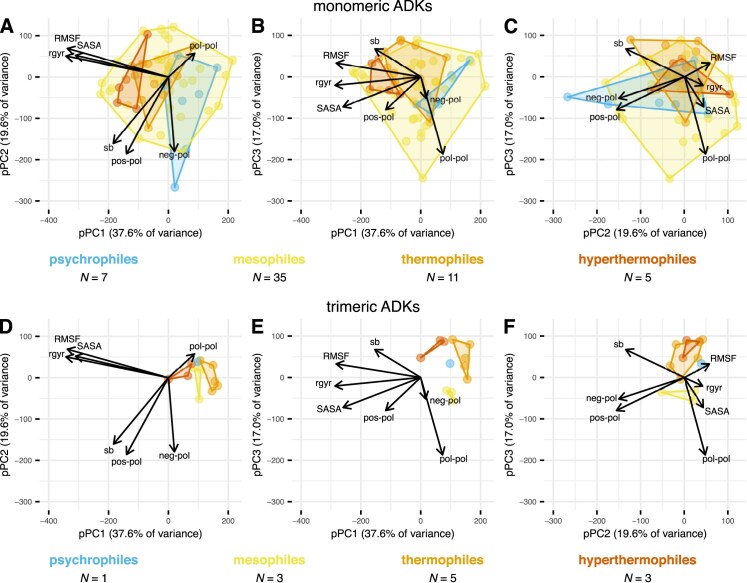
Phylogenetic principal component analysis of 7 structural variables (arrows) for the 70 prokaryotic adenylate kinases (ADKs) of this study. RMSF stands for the root mean square fluctuation of alpha carbon atoms and is a measure of structural flexibility. SASA is the solvent-accessible surface area, a proxy of surface compactness. $\text{r}_{\text{gyr}}$ is the radius of gyration of alpha carbon atoms and is a measure of overall structure compactness. The remaining variables represent counts of different contact types between pairs of amino acid side chains: (i) negatively charged–polar (“neg-pol”), (ii) positively charged–polar (“pos-pol”), (iii) polar–polar (“pol-pol”), or (iv) salt bridges (“sb”; contacts between a negatively charged and a positively charged side chain). The four thermal groups are shown in different colors, whereas monomeric (A–C) and trimeric ADKs (D–F) are shown separately for clarity.

### Comparison of the structural parameter space occupied by the four thermal groups

To understand whether thermal adaptation of prokaryotic ADKs involves dramatic shifts in the structural parameter space (our first main question), we conducted 10 replicate molecular dynamics simulations for a combined length of 2 *μ*s per ADK (see the *Methods* section). In these simulations, we specified a different (“native”) temperature for each group, namely 280 K (6.85 °C) for psychrophilic ADKs, 300 K (26.85 °C) for mesophilic ADKs, 330 K (56.85 °C) for thermophilic ADKs, or 355 K (81.85 °C) for hyperthermophilic ADKs. The simulations yielded estimates of seven structural variables that describe the flexibility, compactness, and presence or absence of amino acid contacts per ADK (see the caption of Figure 4 for a description of each variable), which we analyzed through phylogenetic principal component analysis (pPCA).

Overall, the four thermal groups overlapped considerably throughout the parameter space, with the most pronounced separation observed in the first phylogenetic principal component (pPC1) where psychrophiles and hyperthermophiles had no overlap (Figure 4). Nevertheless, mesophiles occurred throughout the entire range of pPC1, with values exceeding those of both psychrophiles and hyperthermophiles. The variables that most strongly correlated with pPC1 reflected structural flexibility (root mean square fluctuation [RMSF]) and compactness (solvent-accessible surface area [SASA] and radius of gyration [$\text{r}_{\text{gyr}}$]). Monomeric ADKs (Figure 4A) covered a much wider range of parameter space than their trimeric orthologs (Figure 4B), which may again reflect the structural constraints that trimerization imposes.

These results suggest that (a) there are likely multiple evolutionary paths toward adaptation to extreme thermal environments, (b) such environments may be well tolerated by certain mesophilic ADKs, and (c) changes in flexibility and/or compactness may indeed contribute to the thermal adaptation of prokaryotic ADKs.

### Impacts of temperature on ADK flexibility

The simulations performed so far enable us to infer the interspecific relationship between ADK flexibility (as measured by the RMSF variable) and temperature. To also capture the intraspecific relationship for our second main question, we performed additional simulations for five ADKs at seven more temperatures ranging from 6.85 °C to 94.35 °C (henceforth referred to as “non-native temperatures”). Given that monomeric ADKs occupy a much wider range of parameter space (Figure 4), the five ADKs that we selected were all monomeric. We then fitted a series of candidate models to simultaneously estimate the intra- and interspecific relationships (if any) between RMSF and temperature. In these models, we accounted for (a) the uncertainty around each RMSF estimate, (b) the ADK type (monomeric or trimeric), (c) the LID length (short or long), and/or (d) the evolutionary relationships among species (through a phylogenetic random effect on the intercept). The best-supporting model was identified through model selection (see the *Methods*section).

**Figure 5.**
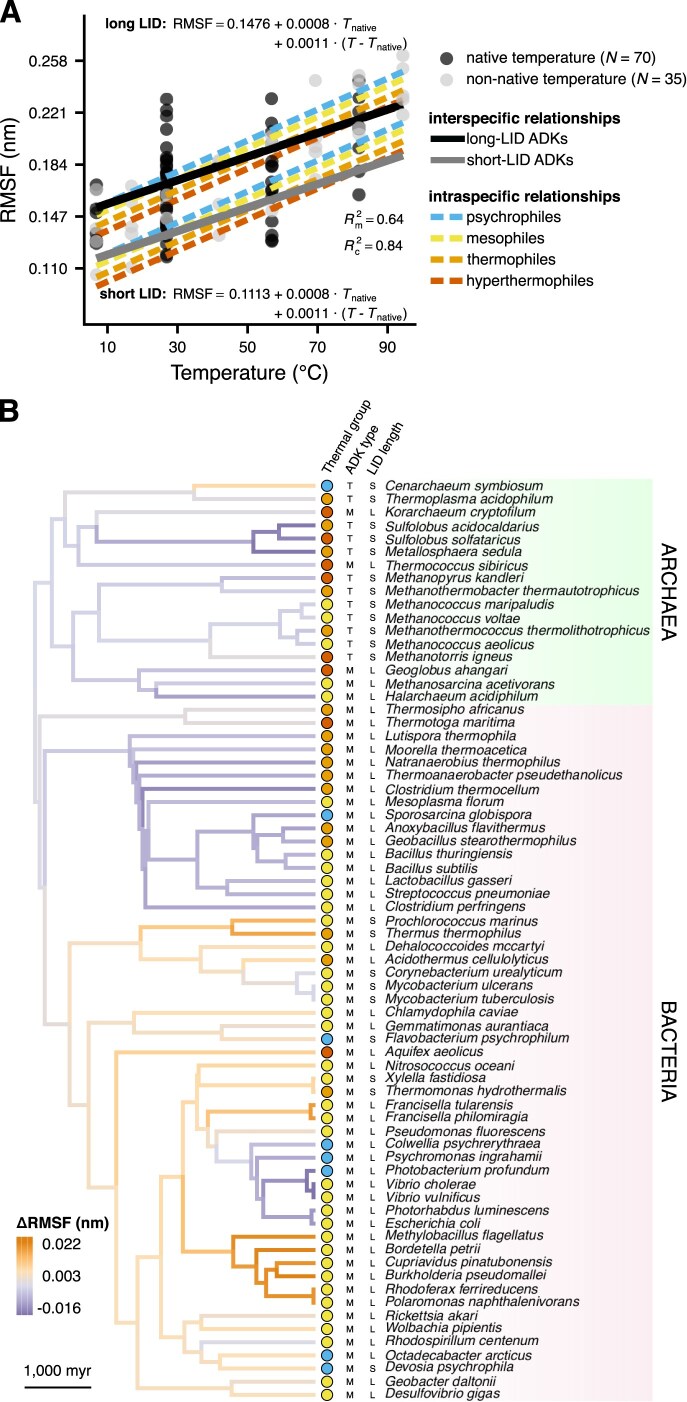
The relationship between root mean square fluctuation (RMSF; a measure of structural flexibility) and temperature. (A) Flexibility increases with temperature, across and within species. Note that intraspecific relationships (dashed lines) are plotted separately for long-LID (top) and short-LID adenylate kinases (ADKs; bottom). (B) Distribution of the phylogenetic random effect on the intercept across the branches of the species’ phylogeny. Each branch is colored based on its difference from the intercept of the corresponding relationship in panel A. Panel B also shows the thermal group for each species, the ADK type (M for monomeric, T for trimeric), and the LID length (S for short, L for long).

At the within-species level, we found that flexibility (RMSF values) consistently increased with temperature (Figure 5A; Figure S4), as expected from the increase in the total energy of the system at higher temperatures. In addition, the interspecific temperature slope was not zero (as the corresponding states hypothesis predicts; Figure 1), but rather positive, with long-LID and short-LID ADKs differing in their intercepts (see also Table S2). The aforementioned factors explained 64% of variance in RMSF ($R^2_{\text{m}}$), whereas a phylogenetic random effect on the intercept captured an additional 20% of variance ($R^2_{\text{c}}$). This suggests that evolutionary shifts in flexibility can also arise in response to other—phylogenetically structured—factors (e.g., the range of pH values tolerated by each species). In particular, while almost all archaea in our dataset appear to have evolved low temperature- and LID-corrected RMSF values, bacterial clades are split between high- and low-RMSF groups (Figure 5B).

### Impacts of temperature on ADK compactness

**Figure 6.**
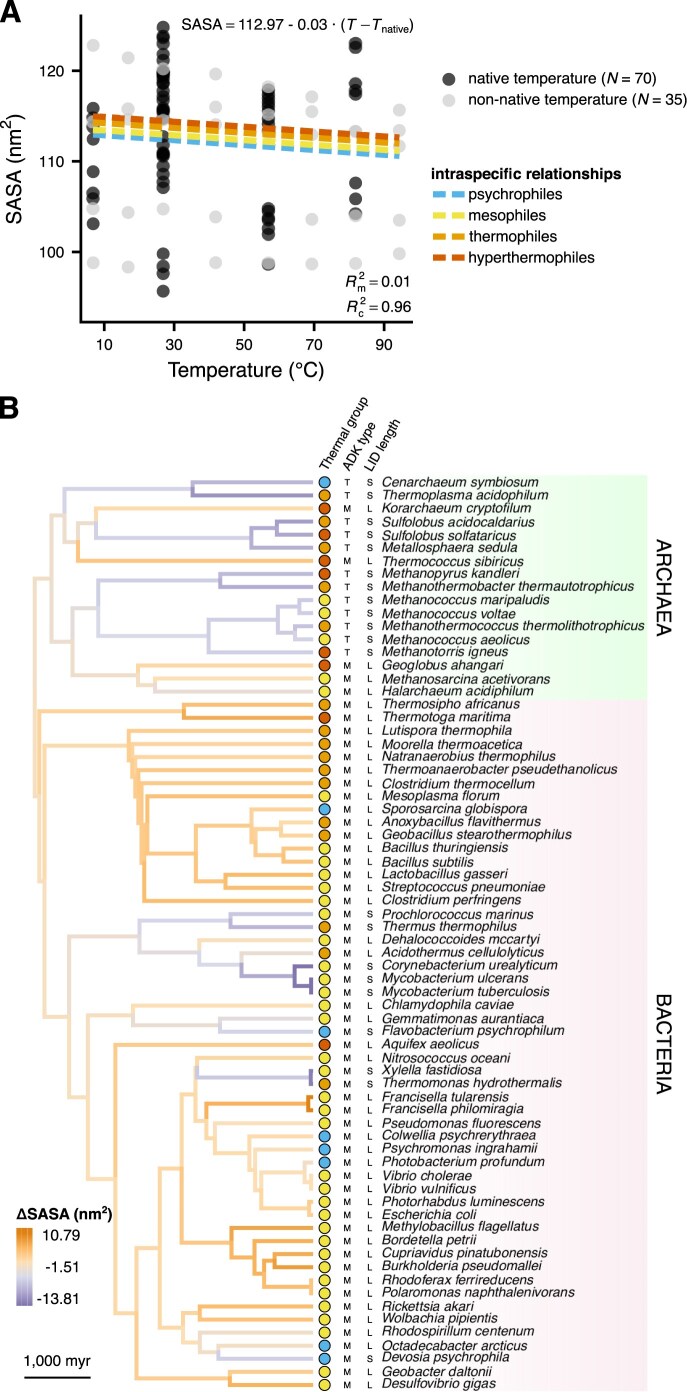
The relationship between solvent-accessible surface area (SASA) and temperature. (A) While SASA statistically decreases with temperature within species, this accounts for only 1% of the variance. (B) Distribution of the phylogenetic random effect on the intercept across the branches of the species’ phylogeny, as in Figure 5.

Following a similar approach to that in the previous subsection, we detected a statistically supported, albeit very weak intraspecific decline in SASA (a surface compactness proxy) with temperature (Figure 6A). In contrast, a systematic interspecific relationship was not supported (Table S3). Temperature explained a negligible amount of variance in SASA (1%), with the overwhelming majority (95%) being captured by the phylogenetic random effect on the intercept. We verified the low explanatory power of temperature for SASA by estimating the intraspecific relationship separately for each of the five species for which simulations were performed at multiple temperatures. This showed that SASA systematically decreased with temperature for only three out of five species, with this decrease being non-negligible for only two species (Figure S5). The two species with the steepest intraspecific slopes (*Aquifex aeolicus*, a hyperthermophile and *Sporosarcina globispora*, a psychrophile) lie on opposite ends of the temperature spectrum, which prevents us from drawing any further mechanistic conclusions. Examining the phylogenetic distribution of the estimated random effect revealed that almost all bacterial lineages had evolved relatively high SASA values, in contrast to archaeal lineages, many of which had shifted toward lower SASA (Figure 6B).

**Figure 7.**
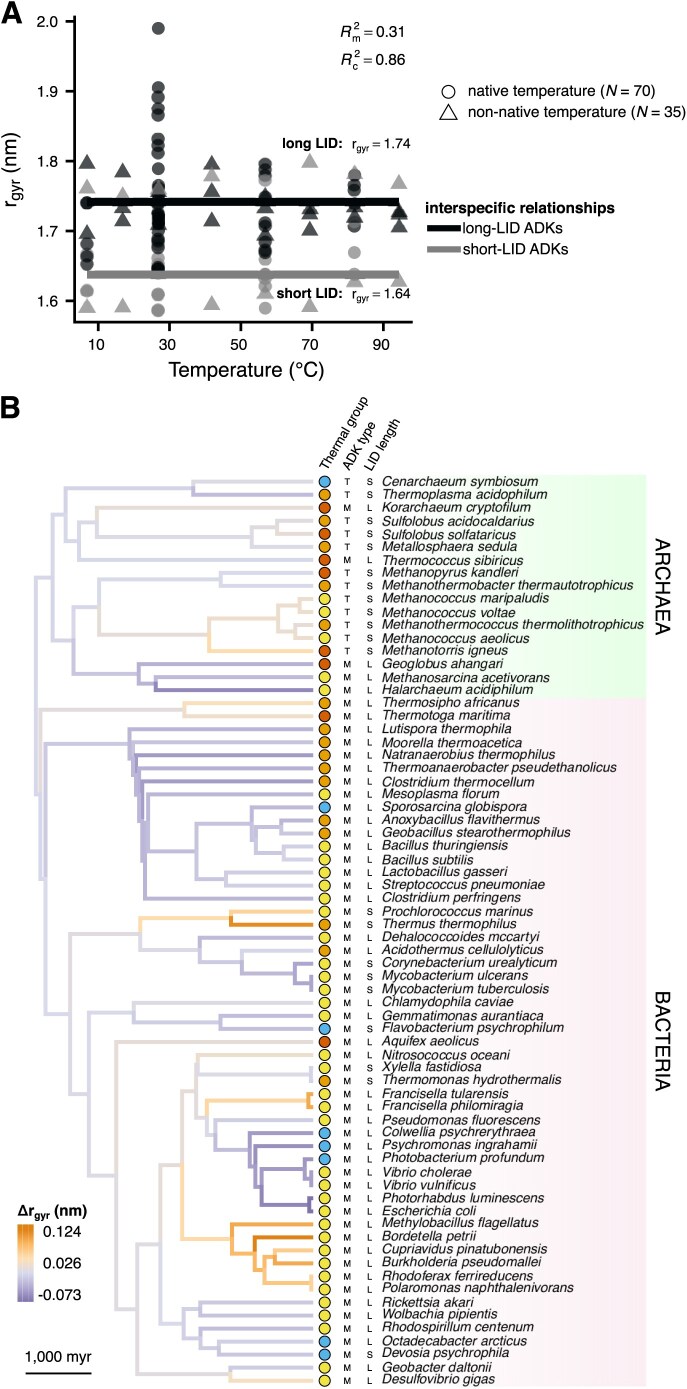
The relationship between the radius of gyration (a measure of overall structure compactness) and temperature. (A) Temperature does not systematically affect the radius of gyration, either within or across species. Long- and short-LID adenylate kinases (ADKs) have slightly different intercepts. (B) Distribution of the phylogenetic random effect on the intercept across the branches of the species’ phylogeny, as in Figure 5.

A systematic relationship between temperature and $\text{r}_{\text{gyr}}$ (a measure of overall structure compactness) could not be found, either within or across species (Figure 7; Figure S6 and Table S4). Long- and short-LID ADKs differed in $\text{r}_{\text{gyr}}$ by 0.1 nm on average, which explained 31% of variance. 55% of the remaining variance was captured by a phylogenetic random effect on the intercept. These results indicate that neither acute nor long-term exposure to elevated temperatures systematically influences the overall compactness of prokaryotic ADKs.

### Amino acid contacts and thermal adaptation

**Figure 8.**
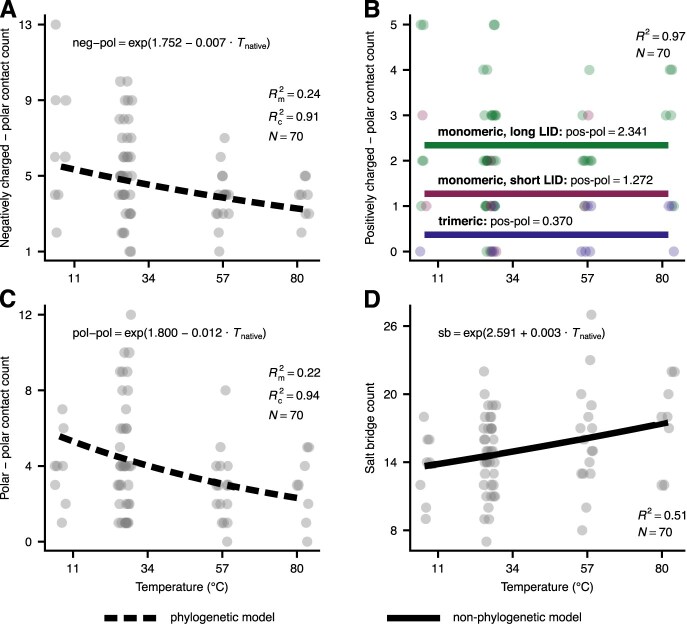
The impact of temperature on the counts of the four contact types. Each data point corresponds to a different adenylate kinase (ADK), whereas the lines represent the best-fitting model for each panel (see the *Methods* section). In panel B, different colors are used for trimeric ADKs, monomeric ADKs with a short LID, and monomeric ADKs with a long LID. To reduce the extensive overlap among data points (especially in panel B), we have slightly displaced data points along the horizontal axis. See also Figures S2 and S3 for the distributions of the phylogenetic random effects of the models shown in panels A and C.

Our third key question was whether contacts among amino acids play a major role in the adaptation of ADKs to different thermal environments. We addressed this question in two ways. First, we examined whether there is a systematic interspecific relationship between contact counts and temperature, separately for each contact type (see Figure 4). We found that adaptation to higher temperatures is weakly associated with a small increase in the number of salt bridges (Table S8) and slight declines in negatively charged–polar (Table S5) and polar–polar contact counts (Table S7). Specifically, compared to psychrophilic ADKs, hyperthermophilic orthologs have on average three more salt bridges, two fewer negatively charged–polar contacts, and three fewer polar–polar contacts (Figure 8). In contrast, the number of positively charged–polar contacts was independent of temperature (Table S6). It is worth pointing out here that these relationships were quite noisy, with temperature explaining between 22% and 51% of variance.

**Figure 9.**
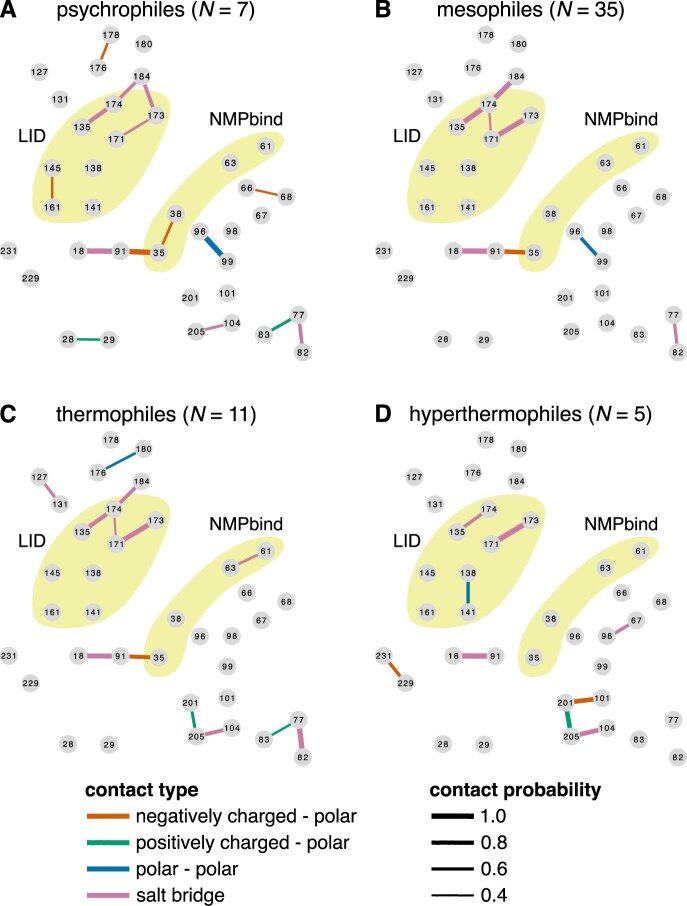
Average contact networks for each thermal group. Each node corresponds to a different column of the amino acid alignment of monomeric adenylate kinases (ADKs). Nodes are positioned approximately where they would lie along the ADK structure (see Figure 2), with the LID and NMPbind domains shown in yellow. The four contact types are also shown in different colors, with line thickness being proportional to the probability of each contact. Contacts with a probability below 0.4 are not shown.

Second, we investigated whether thermal adaptation may be linked to the presence or absence of several key contacts at specific regions of the protein. For the latter, we inferred the average contact network for the monomeric ADKs of each thermal group. More precisely, for all possible pairs of alignment columns, we estimated the probability of the presence of a contact while accounting for the evolutionary relationships among species. We then built networks that included all contacts with a probability of at least 0.4 (Figure 9). Overall, we detected significant within-group variation in the presence and absence of amino acid contacts, with very few contacts having probabilities close to 1 within a given thermal group (see also Figure S7, where we raised the probability cutoff from 0.4 to 0.7). It is worth noting that $\sim 93.5$% of all observed contacts had a probability below 0.4 and, hence, are not shown in the average networks of Figure 9. Furthermore, most of the commonly occurring contacts were either shared by all four thermal groups (e.g., salt bridges in the LID domain) or only shared by nonadjacent thermal groups (e.g., the positively charged–polar contact between alignment columns 77 and 83 of psychrophiles and thermophiles).

These findings indicate that adaptation of ADKs to extreme low- or high-temperature environments is not tightly linked to the presence or absence of specific contacts among amino acid side chains. Instead, different lineages appear to respond to similar thermal challenges with very different evolutionary solutions.

## Concluding remarks

This study shows that thermal adaptation of prokaryotic adenylate kinases (ADKs) is achieved at the structural level in diverse ways (Figure 4). In particular, we found evidence for a systematic increase in global structural flexibility with temperature, both within species (as expected; see Figure 1) and across species (Figure 5). This means that psychrophilic orthologs are on average (a) more flexible than their warmer-adapted orthologs at any given temperature and (b) less flexible than their warmer-adapted orthologs when compared at their respective native temperature ranges. The aforementioned pattern does not support the “corresponding states” hypothesis (Figure 1), which would predict an interspecific slope of zero. In contrast to structural flexibility, a change in structural compactness, either only at the solvent-exposed surface area of the protein or overall, could not be systematically linked to thermal adaptation (Figures 6 and 7). Similarly, the topology and types of contacts formed among pairs of amino acids varied considerably across ADKs from similar thermal environments (Figures 8 and 9). Together, our results suggest that the thermal adaptation of prokaryotic ADKs is not mainly driven by a highly conserved set of structural modifications, but by numerous lineage-specific mechanisms.

The patterns observed in our study are consistent with the concept of “historical contingency” *sensu* Gould (1989). According to this concept, the outcome of a given evolutionary change on a lineage strongly depends on the order of historical events (e.g., mutations, environmental changes) that the lineage has experienced. As evolutionary trajectories diverge, possible paths to a given functional innovation (e.g., the ability of an enzyme to operate at high temperatures) are continuously reshaped, changing the accessibility of the innovation (see, e.g., Blount et al., 2018; Harms & Thornton, 2014; Park et al, 2022; Starr & Thornton, 2016; Storz, 2016). At the molecular level, historical contingency implies that a mutation that would enable a particular ADK to tolerate high temperatures will not necessarily have the same effect on other orthologs, due to differences in amino acid sequence, which give rise to different patterns of epistasis. Furthermore, a given mutation may simultaneously impact multiple characteristics of an enzyme (e.g., flexibility, compactness, activity), generating a vast diversity of outcomes in different lineages (Ogbunugafor et al., 2023), some of which may be strongly deleterious and, hence, eliminated. Subsequent mutations would further increase the complexity, dramatically reducing the predictability of mutational fitness effects across large phylogenetic distances. This evolutionary mechanism likely explains the remarkable variation in the occurrence of amino acid contacts within each thermal group (Figure 9). Historical contingency also extends to the level of the entire protein structure, given the phylogenetic structure exhibited by RMSF, SASA, and $r_{\text{gyr}}$. Thus, we can conclude that the thermal adaptation of prokaryotic ADKs appears to be constrained by historical contingency at multiple levels.

A limitation of our study is that we did not include any kinetic variables in our analyses, i.e., $k_{\text{cat}}$ (turnover number; the catalytic velocity of a single enzyme under saturating substrate conditions), $K_{\text{M}}$ (the Michaelis constant; a measure of substrate binding affinity), and their ratio (a measure of catalytic efficiency). Such variables would enable us to test for a trade-off between structural stability and catalytic activity, as has been previously suggested (e.g., Maffucci et al., 2020; Miller, 2017; Nguyen et al., 2017; Stark et al., 2022). Unfortunately, published estimates of these parameters for prokaryotic ADKs are largely unavailable. Nevertheless, a recent study by Muir et al. (2025) generated a dataset of kinetic parameters for 193 prokaryotic ADKs, 17 of which were also included in our dataset. Across those 17 ADKs, there was no systematic relationship between temperature-normalized flexibility and kinetic parameters (Figure S8). This pattern suggests the absence of a stability-activity trade-off in prokaryotic ADKs, in line with the conclusions of Muir et al. (2025). Given the importance of ADK for cellular energy homeostasis and, hence, fitness (Couñago & Shamoo, 2005; Couñago et al., 2006, 2008; Peña et al., 2010; Tükenmez et al., 2016), the lack of this trade-off may arise from strong selection for sufficient ADK activity in diverse thermal environments. In any case, a possible link between the strength of the stability-activity trade-off and the importance of a given enzyme for fitness remains to be explored in future studies.

It is worth noting that the adaptation of enzyme structures is just one way in which species respond to thermal challenges. Other mechanisms may include (a) evolving multiple gene transcripts or gene copies with different thermal characteristics (Sanchez-Perez et al., 2008; Telonis-Scott et al., 2014), (b) tweaking the expression levels of heat shock proteins (Oksala et al., 2014), (c) changes in metabolic networks (Braakman et al., 2017), (d) modifications in the fluidity of cellular membranes (Ernst et al., 2016), and (e) behavioral responses for locating more thermally suitable (micro-)habitats (Freitas et al., 2015; Ramalho et al., 2023). Such mechanisms are not mutually exclusive, with their relative importance varying across species, depending on their physiological, morphological, or other traits and the characteristics of their local environment.

The macroevolutionary patterns described in this study share several similarities with those of previous studies, focusing on the macroevolution of ectotherms’ physiological and ecological responses to temperature. Such responses are typically unimodal and governed by a number of parameters, which also exhibit phylogenetic structure—to various extents—when examined over timescales of several millions of years (e.g., see Bennett et al., 2021; Bodensteiner et al., 2021; Kontopoulos et al., 2020a, b; Pawar et al., 2024; Phillips et al., 2022; Qu & Wiens, 2020; Smith et al., 2022). Some commonly reported patterns for physiological and ecological thermal responses are that (a) their thermal optima are strongly phylogenetically heritable (Kontopoulos et al., 2020a, b; Pawar et al., 2024; Smith et al., 2022), (b) their thermal sensitivity below the optimum is less phylogenetically heritable and can exhibit bursts of rapid evolution (Kontopoulos et al., 2020a, b; Phillips et al., 2022), and (c) their lower thermal limit usually has a higher evolutionary rate than its upper counterpart (Bennett et al., 2021; Bodensteiner et al., 2021; Qu & Wiens, 2020). Although some studies have reported associations between the thermal optima of physiological rates and those of individual enzymes (or their melting temperatures; see e.g., Engqvist, 2018; Stark et al., 2022), a general mechanistic understanding of how macroevolutionary patterns at the enzyme level influence those at higher levels of biological organization remains elusive.

Overall, our study provides quantitative estimates of the intra- and interspecific relationships between environmental temperature and several structural variables of prokaryotic ADKs, as well as any phylogenetically structured deviations from such relationships (e.g., see Figure 5B). Future studies could focus on obtaining similar estimates for many other enzymes from diverse taxonomic groups, which would ultimately pave the way for systematic, genome-wide comparisons of the relative importance of different structural modifications for the thermal adaptation of enzymes.

## Methods

### Compilation of the dataset of ADK structures

The ADKs included in this study were chosen between October 2016 and February 2018. At that time, there were fewer than 20 species of bacteria and archaea with an experimentally determined ADK structure available in the Protein Data Bank. Thus, to obtain a larger and more diverse dataset, we manually selected further ADK sequences from the UniProt database (The UniProt Consortium, 2017), representing a wide range of clades and thermal environments. To estimate the structure of each ADK sequence, we performed homology modeling with the Phyre2 server (Kelley et al., 2015). This approach uses structural information from one or more experimentally determined ADKs (templates) to produce structural models for ADKs for which only the sequence is available (queries). More specifically, we utilized the intensive mode of the Phyre2 server, which automatically selects the most suitable template(s) for each query sequence based on the confidence and coverage of the sequence alignment between the query and template(s). This method also incorporates an *ab initio* step to model amino acids that are not covered by the template(s). To ensure that the quality of all resulting models was sufficiently high, we ensured that all 70 ADKs had no more than four non-covered amino acids and performed additional quality evaluation tests (Figure S1).

### Estimation of evolutionary trees

#### Species tree

To obtain a time-calibrated phylogeny for the species in our ADK dataset, we extracted a tree topology—that included all 70 species—from the Open Tree of Life (OTL; Hinchliff et al., 2015), and a time-calibrated phylogeny—for a subset of them—from the TimeTree database (Kumar et al., 2022). We then used the congruify.phylo function of the geiger R package (v. 2.0.11; Pennell et al., 2014) to transfer age estimates from the TimeTree phylogeny to that of the OTL, where possible (Eastman et al., 2013). The remaining nodes were time-calibrated using the treePL software (Smith & O’Meara, 2012).

#### ADK gene tree

To reconstruct the ADK gene tree, we first aligned the 70 amino acid sequences with MAFFT (v. 7.490; Katoh & Standley, 2013) using the G-INS-i global alignment algorithm. We next ran IQ-TREE (v. 2.3.5; Minh et al., 2020) to identify the best-fitting amino acid substitution model, and perform 300 maximum-likelihood tree searches and 300 nonparametric bootstrap replicates. Finally, we mapped bootstrap support values to the maximum-likelihood tree with the highest log-likelihood.

### Molecular dynamics simulations

#### Simulation protocol

Molecular dynamics simulations were conducted with the Desmond software suite and its graphical interface, Maestro (Version 10.6.013, Release 2016-2). Specifically, for each ADK structure, we first capped the N- and C-termini, added missing hydrogen atoms, and performed energy minimization in vacuum under the OPLS 2005 force field (Banks et al., 2005) to remove potential atom clashes. We then solvated the resulting structure in a periodic orthorhombic simulation box with simple point charge water molecules (Berendsen et al., 1981), and with Na^+^ and Cl^−^ ions added for neutralizing the charge of the protein and for generating a salt concentration of 0.15 M. Box volumes ranged between $\sim$207 and $\sim$360 nm^3^, with the median being at $\sim$248 nm^3^. Each system was next relaxed through Desmond’s standard protocol, following which we performed 10 replicate simulations of 200 ns per ADK. In these simulations, the temperature was held constant using the Nosé–Hoover thermostat (Martyna et al., 1992) at a different value per thermal group (see the *Comparison of the structural parameter space occupied by the four thermal groups* subsection). Similarly, we ensured that the pressure of the system was held constant at the default value of 1.01325 bar by enforcing isotropic coupling and a relaxation time of 2 ps using the Martyna–Tobias–Klein barostat (Martyna et al., 1994). For van der Waals and short-range electrostatic interactions, we set the cutoff radius to 0.9 nm. Finally, we set the integration time step to 2 fs (the default value) and recorded a snapshot of the system every 0.5 ns.

We also performed simulations at seven additional temperatures (from 6.85 °C to 94.35 °C) for the ADKs of (a) *Sporosarcina globispora* (a psychrophilic bacterium), (b) *Mycobacterium tuberculosis* (a mesophilic bacterium), (c) *Methanosarcina acetivorans* (a mesophilic archaeon), (d) *Thermus thermophilus* (a thermophilic bacterium), and (e) *Aquifex aeolicus* (a hyperthermophilic bacterium).

All molecular dynamics simulations were executed on desktop machines equipped with CUDA-enabled GPUs (e.g., GeForce GTX TITAN, Tesla P100) for a total time of $\sim$35,300 hours. We would like to note that this runtime is a small fraction of what we would expect if we had performed the simulations on non-GPU systems.

#### Analysis of simulation trajectories

We used the Gromacs 2023 package (Abraham et al., 2015) to calculate the root mean square fluctuation (RMSF) of alpha carbon atoms, the solvent-accessible surface area (SASA), and the radius of gyration of alpha carbon atoms ($\text{r}_{\text{gyr}}$). To measure the number of contacts among amino acid side chains, we used the MDAnalysis Python package (Gowers et al., 2016; Michaud-Agrawal et al., 2011). Specifically, we applied a typically used distance cutoff of 0.5 nm between the centers of the interacting chemical groups of the two amino acids (e.g., carboxyl group in Asp and Glu, or guanidine group in Arg). From the resulting contacts, we excluded those that occurred for less than 50% of simulation time. Finally, we classified contacts based on the properties of the participating amino acids. Specifically, we treated (a) Arg and Lys as positively charged, (b) Asp and Glu as negatively charged, and (c) Asn, Gln, Ser, Thr, and Tyr as polar amino acids.

### Phylogenetic comparative analyses

#### Phylogenetic PCA

We examined the distribution of the 70 ADKs throughout the structural parameter space by applying a phylogenetic PCA (Revell, 2009) to the matrix of structural variables, estimated at one (native) temperature per ADK. More precisely, we used the phyl.pca function of the phytools R package (v. 2.1-1; Revell, 2024), accounting for the level of phylogenetic signal in the data by simultaneously optimizing the $\lambda$ parameter. Given that there was not adequate phylogenetic signal for inferring a statistically robust ADK gene tree (Figure 3E), we instead used the species’ phylogeny as a proxy of the evolutionary relationships among ADKs.

#### Estimation of any intra- and interspecific relationships of RMSF, SASA, and $\text{r}_{\text{gyr}}$ with temperature

To understand if RMSF, SASA, and $\text{r}_{\text{gyr}}$ vary systematically with temperature within or across species, we fitted 32 alternative generalized linear mixed models for each of these three variables with the MCMCglmm R package (v. 2.36; Hadfield, 2010). RMSF, SASA, and $\text{r}_{\text{gyr}}$ were treated as Gaussian-distributed response variables, with the measurement uncertainty of each data point explicitly accounted for, making each model “meta-analytic.” Possible explanatory variables were (a) the native temperature (for the interspecific slope), (b) the difference between the non-native and native temperature (if applicable, for the intraspecific slope), (c) the ADK type (monomeric or trimeric), and (d) the length of the LID (short or long). We fitted models with all possible combinations of these explanatory variables, as well as an empty (intercept-only) model. We also specified a phylogenetic variant of each model by adding a phylogenetic random effect on the intercept. The priors that we used were relatively uninformative, namely the default Gaussian prior for fixed effects, a parameter-expanded prior for the phylogenetic random effect (if applicable), and an inverse Gamma prior for the residual variance. For each model, we executed three independent chains for 1 million generations, with posterior samples obtained every 100 generations after discarding the first 100,000 as burn-in. We ensured that the three chains per model had converged to statistically equivalent posterior distributions and that the latter were sufficiently sampled by verifying that, for each model parameter, (i) the effective sample size was at least 1,000 and (ii) the potential scale reduction factor was below 1.1. To identify the best-fitting model for each response variable, we first excluded models if any of their fixed-effects coefficients (other than the intercept) had a 95% highest posterior density interval that included zero. From the remaining models, we selected the one with the lowest deviance information criterion value (Spiegelhalter et al., 2002). For this model, we calculated the marginal and conditional $R^2$ values following Hadfield and Nakagawa (2010). $R^2_{\text{m}}$ stands for the proportion of variance that is captured by the fixed effects, whereas $R^2_{\text{c}}$ stands for the variance captured by both the fixed and the random effects.

#### Linking contacts to thermal adaptation

To test for any interspecific relationships between contact counts and temperature, we followed the approach described in the previous subsection. The main differences were that the response variables were treated as Poisson-distributed and that we did not attempt to estimate any intraspecific relationships. For the three independent chains to reach convergence, we set the number of MCMC generations to 50 million, the burn-in to 5 million, and sampled from the posterior every 15,000 generations.

For the contact network analysis, we used the amino acid alignment of the 58 monomeric ADKs to objectively compare the observed contacts across ADKs. More precisely, we created a unique identifier for each contact, based on the alignment column numbers of the two participating amino acids. We next constructed matrices where the rows were the 58 ADKs and the columns were the contacts. We made separate matrices for each thermal group and each contact type. The values in these matrices were either 0 (the contact is missing) or 1 (the contact is present). For columns where all values were 0 or 1, the contact probability is necessarily equal to 0 or 1, respectively. For all remaining matrix columns, we fitted a phylogenetic threshold model (Felsenstein, 2005; Hadfield, 2015) with MCMCglmm, separately for each thermal group. This type of model assumes that the discrete response variable (the presence/absence vector of a given contact in this case) is governed by an unobserved continuous variable, called the “liability.” The contact should be absent if the liability is below 0 and present otherwise. To this end, each threshold model had the presence/absence vector as the response variable, no predictors other than an intercept, and a phylogenetic random effect on the intercept. The priors were set similarly to those in the previous subsection, except for the residual variance, which is not identifiable in threshold models and had to be fixed to 1. We executed the three chains per model for 500,000 generations, removed samples from the first 50,000 generations as burn-in, and then sampled from the posterior every 50 generations. After running convergence and sampling diagnostics as previously described, we calculated the contact probability based on the posterior distribution of the intercept. Because the intercept stands for the phylogenetically corrected liability for a given contact and thermal group, the contact probability can be calculated as the number of posterior samples of the intercept with a value of 0 or higher, divided by the total number of samples.

## Supplementary Material

qraf026_Supplemental_File

## Data Availability

The data underlying this article are available from Figshare at https://doi.org/10.6084/m9.figshare.28436891 (Kontopoulos et al., 2025b). The source code for the analyses of this study is available from Codeberg at https://codeberg.org/dgkontopoulos/Kontopoulos_et_al_evolution_of_ADK_structures_2025 and archived on Zenodo at https://doi.org/10.5281/zenodo.14891812 (Kontopoulos et al., 2025a).
