# Supplementary material for: Changes in flexibility but not in compactness underlie the thermal adaptation of prokaryotic adenylate kinases
Source: Evol Lett. 2025 Aug 6;9(5):598–609. doi: 10.1093/evlett/qraf026 (PMC12492129; doi:10.1093/evlett/qraf026)
Supplement: qraf026_Supplemental_File [file qraf026_supplemental_file.pdf]

Supplementary Information for

Changes in flexibility but not in compactness underlie the thermal adaptation of prokaryotic adenylate kinases

Dimitrios - Georgios Kontopoulos[1,2,3,4]✉, Ilias Patmanidis[5,6], Timothy G. Barraclough[1,7], and Samraat Pawar[1]

[1] Department of Life Sciences, Imperial College London, Silwood Park, Ascot, Berkshire, UK
[2] LOEWE Centre for Translational Biodiversity Genomics, Frankfurt am Main, Germany
[3] Senckenberg Research Institute, Frankfurt am Main, Germany
[4] Present address: Department of Ecology and Evolutionary Biology, University of California, Los Angeles, CA, USA
[5] Institute of Physics, Carl von Ossietzky Universität Oldenburg, Oldenburg, Germany
[6] Chemical and Pharmaceutical Biology, Groningen Research Institute of Pharmacy, University of Groningen, Groningen, the Netherlands
[7] Department of Biology, University of Oxford, Oxford, UK
✉email: dgkontopoulos@gmail.com

Contents

S1 List of ADKs included in the present study 2

S2 Quality evaluation of structural models 4

S3 Relationships between temperature and structural variables 5
- S3.1 RMSF 5
- S3.2 SASA 6
- S3.3 r_{gyr} 6
- S3.4 Number of negatively charged - polar contacts (neg-pol) 6
- S3.5 Number of positively charged - polar contacts (pos-pol) 7
- S3.6 Number of polar - polar contacts (pol-pol) 8
- S3.7 Number of salt bridges (sb) 9

S4 Species-specific relationships between temperature, RMSF, SASA, or r_{gyr} 9

S5 Average contact networks with a probability of at least 0.7 11

S6 Correlations between flexibility and kinetic parameters 11

S1 List of ADKs included in the present study

Table S1: Prokaryotic ADKs analysed and their main characteristics.

Species	UniProt ID	Superkingdom	Phylum	Thermal group	LID	Type
Acidothermus cellulolyticus	A0LRP1	Bacteria	Actinomycetota	thermophilic	long	monomeric
Anoxybacillus flavithermus	B7GJ88	Bacteria	Bacillota	thermophilic	long	monomeric
Aquifex aeolicus	O66490	Bacteria	Aquificota	hyperthermophilic	long	monomeric
Bacillus subtilis	P16304	Bacteria	Bacillota	mesophilic	long	monomeric
Bacillus thuringiensis	A0R8K1	Bacteria	Bacillota	mesophilic	long	monomeric
Bordetella petrii	A9IQ39	Bacteria	Pseudomonadota	mesophilic	long	monomeric
Burkholderia pseudomallei	Q3JVB1	Bacteria	Pseudomonadota	mesophilic	long	monomeric
Cenarchaeum symbiosum	A0RUE3	Archaea	Nitrososphaerota	psychrophilic	short	trimeric
Chlamydophila caviae	Q822Z1	Bacteria	Chlamydiota	mesophilic	long	monomeric
Clostridium perfringens	Q0TMR7	Bacteria	Bacillota	mesophilic	long	monomeric
Clostridium thermocellum	A3DJJ3	Bacteria	Bacillota	thermophilic	long	monomeric
Colwellia psychrerythraea	Q47XA8	Bacteria	Pseudomonadota	psychrophilic	long	monomeric
Corynebacterium urealyticum	B1VEX6	Bacteria	Actinomycetota	mesophilic	short	monomeric
Cupriavidus pinatubonensis	Q475G1	Bacteria	Pseudomonadota	mesophilic	long	monomeric
Dehalococcoides mccartyi	Q3Z960	Bacteria	Chloroflexota	mesophilic	long	monomeric
Desulfovibrio gigas	C7U112	Bacteria	Thermodesulfobacteriota	mesophilic	long	monomeric
Devosia psychrophila	A0A0F5PRB6	Bacteria	Pseudomonadota	psychrophilic	short	monomeric
Escherichia coli	P69441	Bacteria	Pseudomonadota	mesophilic	long	monomeric
Flavobacterium psychrophilum	A6GZB9	Bacteria	Bacteroidota	psychrophilic	short	monomeric
Francisella philomiragia	B0TXR2	Bacteria	Pseudomonadota	mesophilic	long	monomeric
Francisella tularensis	Q5NFR4	Bacteria	Pseudomonadota	mesophilic	long	monomeric
Gemmatimonas aurantiaca	C1A6S6	Bacteria	Gemmatimonadota	mesophilic	long	monomeric
Geobacillus stearothermophilus	P27142	Bacteria	Bacillota	thermophilic	long	monomeric
Geobacter daltonii	B9M6F7	Bacteria	Thermodesulfobacteriota	mesophilic	long	monomeric
Geoglobus ahangari	A0A0F7IDB9	Archaea	Methanobacteriota	hyperthermophilic	long	monomeric
Halarchaeum acidiphilum	U3AED7	Archaea	Methanobacteriota	mesophilic	long	monomeric
Korarchaeum cryptofilum	B1L775	Archaea	Thermoproteota	hyperthermophilic	long	monomeric
Lactobacillus gasseri	Q046A5	Bacteria	Bacillota	mesophilic	long	monomeric
Lutispora thermophila	A0A1M6DWR4	Bacteria	Bacillota	thermophilic	long	monomeric
Mesoplasma florum	Q6F1X3	Bacteria	Mycoplasmatota	mesophilic	long	monomeric
Metallosphaera sedula	A4YCZ0	Archaea	Thermoproteota	thermophilic	short	trimeric
Methanococcus aeolicus	A6UW35	Archaea	Methanobacteriota	mesophilic	short	trimeric
Methanococcus maripaludis	A4FXE7	Archaea	Methanobacteriota	mesophilic	short	trimeric
Methanococcus voltae	P43411	Archaea	Methanobacteriota	mesophilic	short	trimeric
Methanopyrus kandleri	Q8TZB0	Archaea	Methanobacteriota	hyperthermophilic	short	trimeric
Methanosarcina acetivorans	Q8TRS3	Archaea	Methanobacteriota	mesophilic	long	monomeric
Methanothermobacter thermautotrophicus	O26135	Archaea	Methanobacteriota	thermophilic	short	trimeric
Methanothermococcus thermolithotrophicus	P43410	Archaea	Methanobacteriota	thermophilic	short	trimeric

Supplementary Table S1 – *Continued from the previous page*

**Species**	**UniProt ID**	**Superkingdom**	**Phylum**	**Thermal group**	**LID**	**Type**
Methanotorris igneus	P43408	Archaea	Methanobacteriota	hyperthermophilic	short	trimeric
Methylobacillus flagellatus	Q1GZB7	Bacteria	Pseudomonadota	mesophilic	long	monomeric
Moorella thermoacetica	Q2RFR8	Bacteria	Bacillota	thermophilic	long	monomeric
Mycobacterium tuberculosis	P9WKF5	Bacteria	Actinomycetota	mesophilic	short	monomeric
Mycobacterium ulcerans	A0PM98	Bacteria	Actinomycetota	mesophilic	short	monomeric
Natranaerobius thermophilus	B2A4G2	Bacteria	Bacillota	thermophilic	long	monomeric
Nitrosococcus oceani	Q3J7A3	Bacteria	Pseudomonadota	mesophilic	long	monomeric
Octadecabacter arcticus	M9RLZ6	Bacteria	Pseudomonadota	psychrophilic	long	monomeric
Photobacterium profundum	Q6LTE1	Bacteria	Pseudomonadota	psychrophilic	long	monomeric
Photorhabdus luminescens	Q7N0P5	Bacteria	Pseudomonadota	mesophilic	long	monomeric
Polaromonas naphthalenivorans	A1VNK2	Bacteria	Pseudomonadota	mesophilic	long	monomeric
Prochlorococcus marinus	A8G742	Bacteria	Cyanobacteriota	mesophilic	short	monomeric
Pseudomonas fluorescens	C3KEC6	Bacteria	Pseudomonadota	mesophilic	long	monomeric
Psychromonas ingrahamii	A1STI3	Bacteria	Pseudomonadota	psychrophilic	long	monomeric
Rhodoferax ferrireducens	Q21TN6	Bacteria	Pseudomonadota	mesophilic	long	monomeric
Rhodospirillum centenum	B6IRS7	Bacteria	Pseudomonadota	mesophilic	long	monomeric
Rickettsia akari	A8GPC9	Bacteria	Pseudomonadota	mesophilic	long	monomeric
Sporosarcina globispora	P84139	Bacteria	Bacillota	psychrophilic	long	monomeric
Streptococcus pneumoniae	B8ZKQ1	Bacteria	Bacillota	mesophilic	long	monomeric
Sulfolobus acidocaldarius	P35028	Archaea	Thermoproteota	thermophilic	short	trimeric
Sulfolobus solfataricus	Q9UX83	Archaea	Thermoproteota	hyperthermophilic	short	trimeric
Thermoanaerobacter pseudethanolicus	B0KCM0	Bacteria	Bacillota	thermophilic	long	monomeric
Thermococcus sibiricus	C6A2Q1	Archaea	Methanobacteriota	hyperthermophilic	long	monomeric
Thermomonas hydrothermalis	A0A1M5AE38	Bacteria	Pseudomonadota	thermophilic	short	monomeric
Thermoplasma acidophilum	Q9HIT1	Archaea	Thermoplasmatota	thermophilic	short	trimeric
Thermosipho africanus	B7IHW7	Bacteria	Thermotogota	thermophilic	long	monomeric
Thermotoga maritima	Q9X1I8	Bacteria	Thermotogota	hyperthermophilic	long	monomeric
Thermus thermophilus	Q5SHQ9	Bacteria	Deinococcota	thermophilic	short	monomeric
Vibrio cholerae	Q9KTB7	Bacteria	Pseudomonadota	mesophilic	long	monomeric
Vibrio vulnificus	Q7MMR5	Bacteria	Pseudomonadota	mesophilic	long	monomeric
Wolbachia pipientis	Q73HA6	Bacteria	Pseudomonadota	mesophilic	long	monomeric
Xylella fastidiosa	B2I757	Bacteria	Pseudomonadota	mesophilic	short	monomeric

S2 Quality evaluation of structural models

We employed two criteria to evaluate the quality of our 70 ADK structures. First, we used the empirical equation of Rost (1999) to examine if the identity percentage between the query and template sequences was sufficiently high relative to the length of their alignment (Fig. S1A). This showed that all query-template alignments fall within the safe homology modelling zone of the parameter space and, therefore, the resulting models are expected to be reliable.

We also used the PROCHECK tool (Laskowski et al., 1993) to generate Ramachandran plots (Branden and Tooze, 2012) and examine the ϕ and ψ backbone dihedral angles of amino acids in each ADK structure. The combinations of these angles can be classified—based on numerous experimentally determined structures from diverse proteins—as being part of a) disallowed regions, b) generously allowed regions, c) additional allowed regions, or d) the most favoured regions. In our case, all ADKs had at least 94.7% of their amino acids in their most favoured and additional allowed regions, whereas the proportion of amino acids in disallowed regions was never higher than 3.2% (Fig. S1B). These results further confirm the high quality of the structural models analysed in this study.

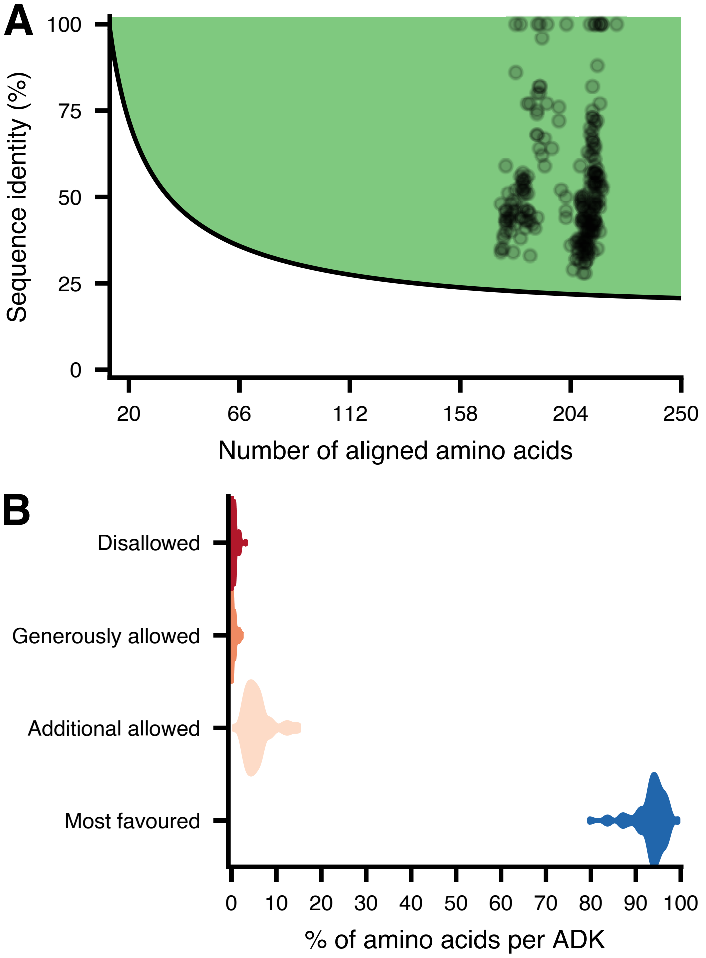

Figure S1: Tests for evaluation of the quality of ADK structures included in this study. (A) The relationship of the sequence identity between template and query sequences against the alignment length. The green area, defined by the empirical equation of Rost (1999), is expected to produce reliable homology models. Note that certain ADKs (those at 100% sequence identity) did not require homology modelling as experimentally determined structures were available. Also, many ADK models were estimated using multiple templates, each of which is shown as a different data point. (B) Distributions of amino acid occurrences at the four regions of Ramachandran plots for all 70 ADKs.

S3 Relationships between temperature and structural variables

S3.1 RMSF

Model	Phylogenetic random effect	Mean DIC
RMSF ∼ Intercept + T_{native} + $(T - T_{\text{native}})$ + LID length	✓	-544.1021
RMSF ∼ Intercept + $(T - T_{\text{native}})$ + LID length	✓	-542.0014
RMSF ∼ Intercept + T_{native} + $(T - T_{\text{native}})$	✓	-539.4008
RMSF ∼ Intercept + T_{native} + $(T - T_{\text{native}})$ + ADK type	✓	-536.2291
RMSF ∼ Intercept + $(T - T_{\text{native}})$	✓	-533.8093
RMSF ∼ Intercept + T_{native} + $(T - T_{\text{native}})$ + LID length	✗	-503.0793
RMSF ∼ Intercept + T_{native} + $(T - T_{\text{native}})$ + ADK type	✗	-479.0617
RMSF ∼ Intercept + T_{native} + $(T - T_{\text{native}})$	✗	-458.0732
RMSF ∼ Intercept + $(T - T_{\text{native}})$ + LID length	✗	-449.4954
RMSF ∼ Intercept + T_{native} + LID length	✓	-436.7387
RMSF ∼ Intercept + T_{native} + LID length	✗	-436.5702
RMSF ∼ Intercept + $(T - T_{\text{native}})$ + ADK type	✗	-433.3798
RMSF ∼ Intercept + $(T - T_{\text{native}})$	✗	-428.3145
RMSF ∼ Intercept + T_{native} + ADK type	✓	-427.9029
RMSF ∼ Intercept + LID length	✓	-427.7656
RMSF ∼ Intercept + LID length	✗	-426.5244
RMSF ∼ Intercept + T_{native}	✓	-423.7988
RMSF ∼ Intercept + T_{native} + ADK type	✗	-423.3583
RMSF ∼ Intercept + ADK type	✓	-418.4271
RMSF ∼ Intercept	✓	-415.7846
RMSF ∼ Intercept + T_{native}	✗	-414.3077
RMSF ∼ Intercept + ADK type	✗	-414.2151
RMSF ∼ Intercept	✗	-409.6807

Table S2: Candidate models for RMSF, listed from best-fitting to worst-fitting. Models with coefficients whose 95% HPD intervals include 0 are not shown.

S3.2 SASA

Model	Phylogenetic random effect	Mean DIC
SASA ∼ Intercept + $(T - T_{\text{native}})$	✓	472.8042
SASA ∼ Intercept + $(T - T_{\text{native}})$ + ADK type	✓	474.1646
SASA ∼ Intercept + T_{native} + ADK type	✓	480.7414
SASA ∼ Intercept + $(T - T_{\text{native}})$ + LID length	✓	482.2283
SASA ∼ Intercept	✓	486.4846
SASA ∼ Intercept + ADK type	✓	487.4268
SASA ∼ Intercept + LID length	✓	490.9502
SASA ∼ Intercept + $(T - T_{\text{native}})$ + ADK type + LID length	✗	553.6843
SASA ∼ Intercept + T_{native} + $(T - T_{\text{native}})$ + LID length	✗	557.1076
SASA ∼ Intercept + $(T - T_{\text{native}})$ + LID length	✗	559.2383
SASA ∼ Intercept + T_{native} + ADK type + LID length	✗	560.4600
SASA ∼ Intercept + T_{native} + LID length	✗	563.9141
SASA ∼ Intercept + ADK type + LID length	✗	568.7994
SASA ∼ Intercept + LID length	✗	574.5256
SASA ∼ Intercept + $(T - T_{\text{native}})$ + ADK type	✗	711.7906
SASA ∼ Intercept + ADK type	✗	714.7713
SASA ∼ Intercept	✗	724.7923

Table S3: Candidate models for SASA, listed from best-fitting to worst-fitting. Models with coefficients whose 95% HPD intervals include 0 are not shown.

S3.3 r_{gyr}

Model	Phylogenetic random effect	Mean DIC
r_{gyr} ∼ Intercept + LID length	✓	-372.9937
r_{gyr} ∼ Intercept	✓	-370.9737
r_{gyr} ∼ Intercept + T_{native} + LID length	✗	-283.4505
r_{gyr} ∼ Intercept + $(T - T_{\text{native}})$ + LID length	✗	-279.3147
r_{gyr} ∼ Intercept + LID length	✗	-276.2915
r_{gyr} ∼ Intercept + T_{native} + ADK type	✗	-261.9447
r_{gyr} ∼ Intercept + $(T - T_{\text{native}})$ + ADK type	✗	-258.7939
r_{gyr} ∼ Intercept + ADK type	✗	-255.8266
r_{gyr} ∼ Intercept	✗	-247.2227

Table S4: Candidate models for r_{gyr}, listed from best-fitting to worst-fitting. Models with coefficients whose 95% HPD intervals include 0 are not shown.

S3.4 Number of negatively charged - polar contacts (neg-pol)

Model	Phylogenetic random effect	Mean DIC
neg-pol ∼ Intercept + T_{native}	✓	304.5047
neg-pol ∼ Intercept	✓	305.5843
neg-pol ∼ Intercept + T_{native}	✗	308.4038
neg-pol ∼ Intercept	✗	316.2293

Table S5: Candidate models for the number of negatively charged - polar contacts, listed from best-fitting to worst-fitting. Models with coefficients whose 95% HPD intervals include 0 are not shown.

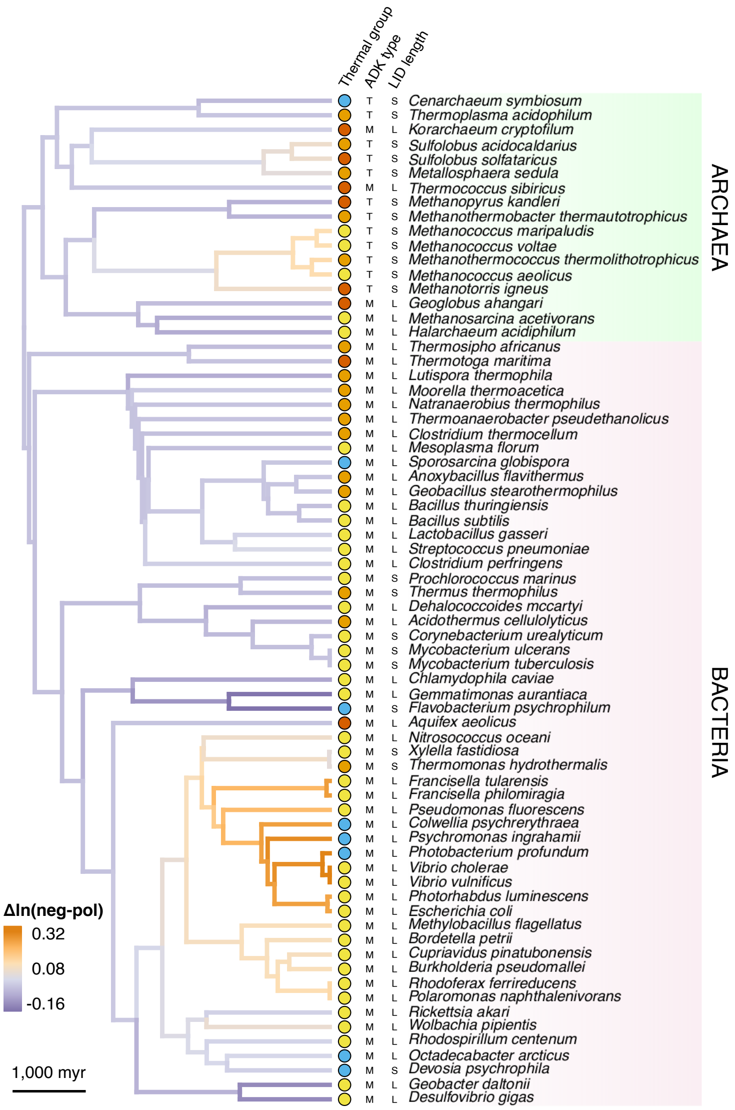

Figure S2: Distribution of the phylogenetic random effect on the intercept of the best-fitting model for negatively charged - polar contact counts (Fig. 8A in the main text) across the branches of the species' phylogeny.

S3.5 Number of positively charged - polar contacts (pos-pol)

Model	Phylogenetic random effect	Mean DIC
pos-pol ∼ Intercept + ADK type + LID length	✗	214.6132
pos-pol ∼ Intercept + ADK type	✗	216.7225
pos-pol ∼ Intercept + LID length	✗	217.8932
pos-pol ∼ Intercept + ADK type	✓	218.1908
pos-pol ∼ Intercept + LID length	✓	219.4394
pos-pol ∼ Intercept	✓	231.2781
pos-pol ∼ Intercept	✗	237.4459

Table S6: Candidate models for the number of positively charged - polar contacts, listed from best-fitting to worst-fitting. Models with coefficients whose 95% HPD intervals include 0 are not shown.

S3.6 Number of polar - polar contacts (pol-pol)

Model	Phylogenetic random effect	Mean DIC
pol-pol ∼ Intercept + T_{native}	✓	298.3921
pol-pol ∼ Intercept	✓	303.0924
pol-pol ∼ Intercept + T_{native}	✗	312.5438
pol-pol ∼ Intercept	✗	313.1715

Table S7: Candidate models for the number of polar - polar contacts, listed from best-fitting to worst-fitting. Models with coefficients whose 95% HPD intervals include 0 are not shown.

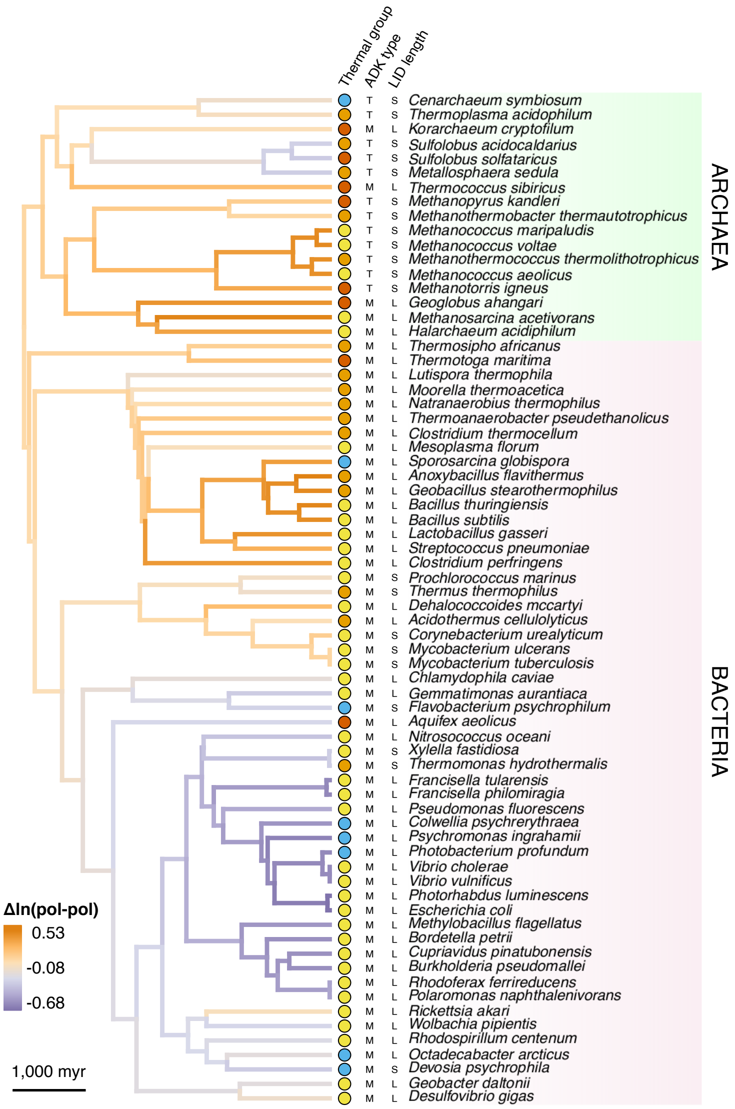

Figure S3: Distribution of the phylogenetic random effect on the intercept of the best-fitting model for polar - polar contact counts (Fig. 8C in the main text) across the branches of the species' phylogeny.

S3.7 Number of salt bridges (sb)

Model	Phylogenetic random effect	Mean DIC
sb ∼ Intercept + T_{native}	✗	385.8649
sb ∼ Intercept + T_{native}	✓	386.5817
sb ∼ Intercept	✗	389.4402
sb ∼ Intercept	✓	389.8820

Table S8: Candidate models for the number of salt bridges, listed from best-fitting to worst-fitting. Models with coefficients whose 95% HPD intervals include 0 are not shown.

S4 Species-specific relationships between temperature, RMSF, SASA, or $\mathbf{r_{gyr}}$

To better compare the effects of temperature on RMSF, SASA, and r_{gyr} among the 5 ADKs for which simulations were performed at 8 temperatures, we fitted variants of the following model with MCMCglmm:

$$\mathrm{Y} = \alpha + \beta \cdot T. \tag{S1}$$

Here, Y is one of RMSF, SASA, or r_{gyr}, α is the intercept, and β the slope of the relationship. We fitted this model separately to each ADK by executing three Markov chains for 100,000 generations. The first 10,000 generations were treated as burn-in and were removed, following which, posterior samples were obtained every 50 generations. We verified that the three chains had converged to statistically equivalent posterior distributions and that posterior sampling was sufficient, as described in the Methods section of the main text. The resulting estimates are shown in Figs. S4, S5, and S6.

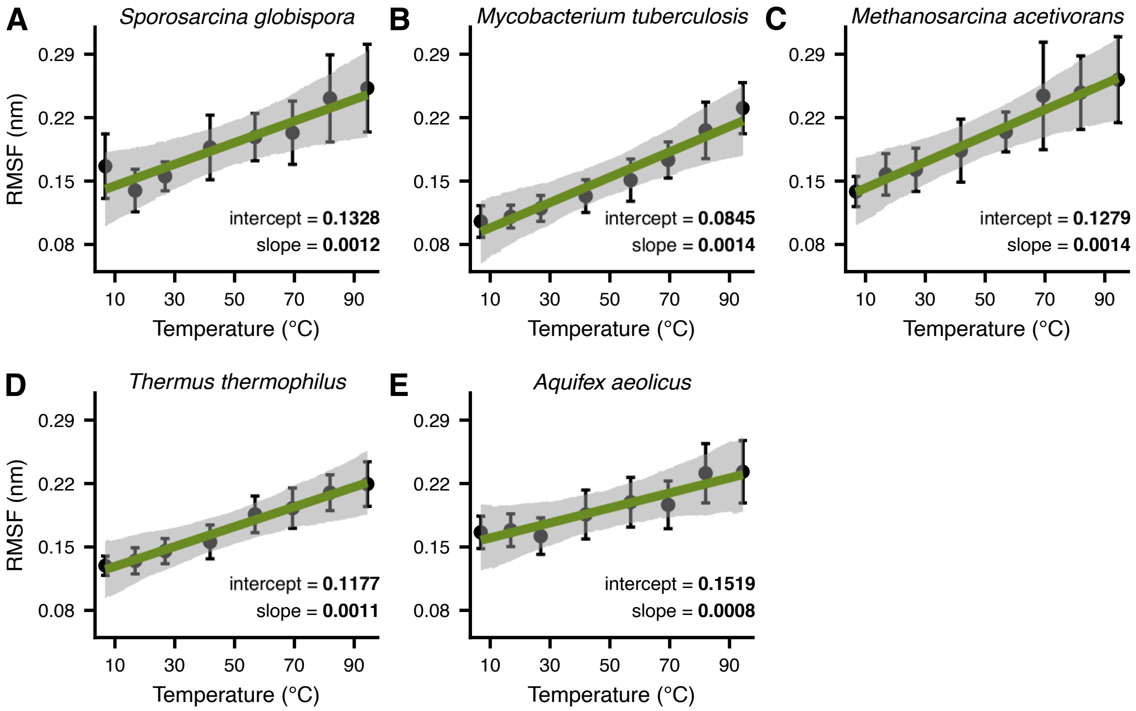

Figure S4: Species-specific relationships between RMSF and temperature. Vertical bars stand for the 95% confidence interval of each data point, whereas grey areas show the 95% Highest Posterior Density (HPD) interval of each fit. Coefficients in bold have a 95% HPD interval that does not include 0.

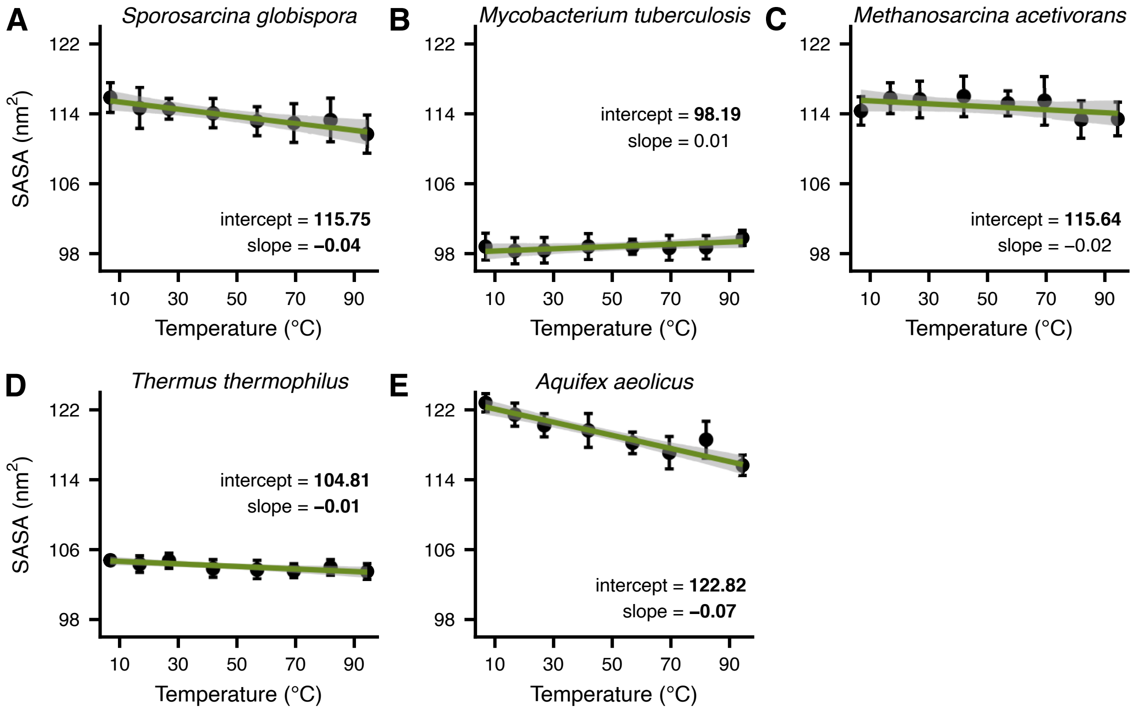

Figure S5: Species-specific relationships between SASA and temperature. Vertical bars stand for the 95% confidence interval of each data point, whereas grey areas show the 95% HPD interval of each fit. Coefficients in bold have a 95% HPD interval that does not include 0.

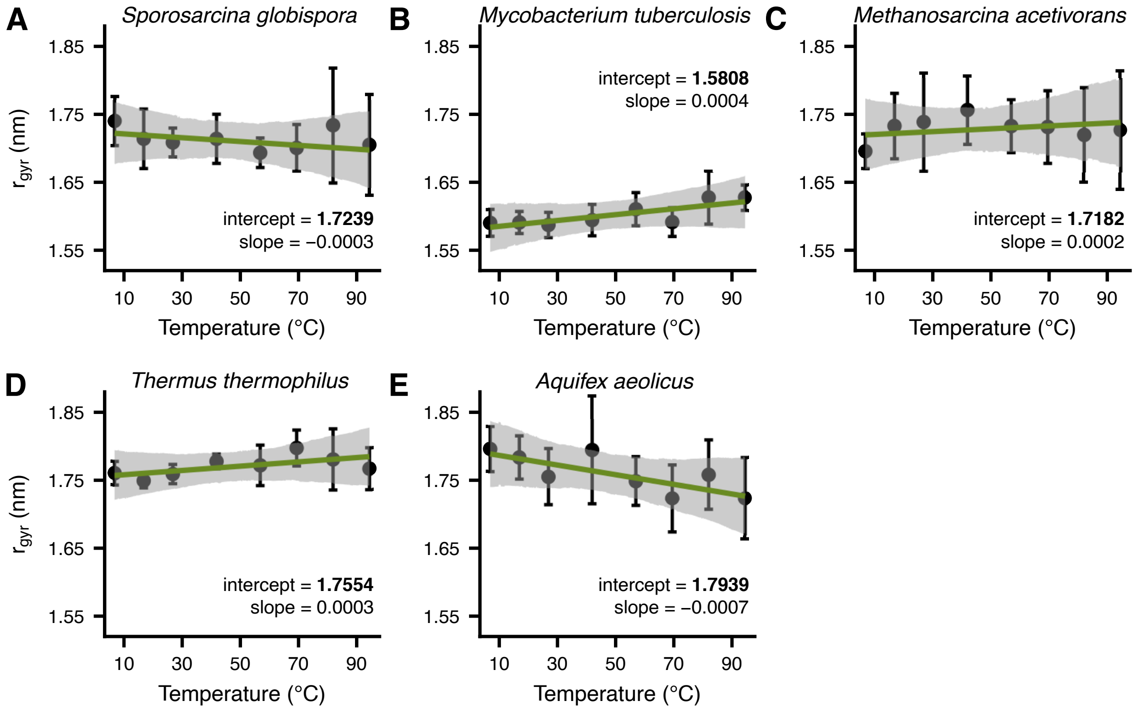

Figure S6: Species-specific relationships between r_{gyr} and temperature. Vertical bars stand for the 95% confidence interval of each data point, whereas grey areas show the 95% HPD interval of each fit. Coefficients in bold have a 95% HPD interval that does not include 0.

S5 Average contact networks with a probability of at least 0.7

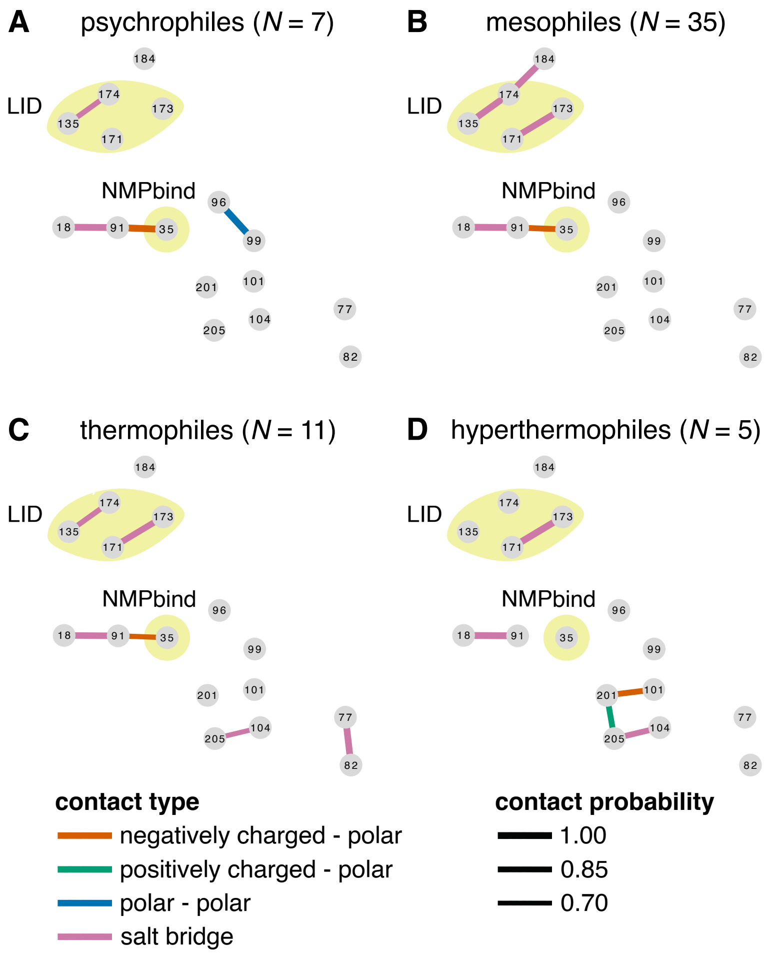

Figure S7: Average contact networks for each thermal group, as in Fig. 9 in the main text. Contacts with a probability below 0.7 are not shown.

S6 Correlations between flexibility and kinetic parameters

We examined whether structural flexibility systematically correlates with kinetic parameters (k_{cat}, K_M, and k_{cat}/K_M) using the recently released dataset of Muir et al. (2025). Their dataset and ours had 17 species in common. Given that their measurements had been obtained at 25°C, we calculated the RMSF value for each of the 17 species at 25°C based on the model shown in Fig. 5 in the main text, including the species-specific random effect on the intercept. We also extracted the uncertainty around each RMSF estimate from the posterior distribution of our model fit.

To infer the correlations, we fitted three models (one for each kinetic parameter) with MCMCglmm. Each of these models had two covarying response variables (temperature-corrected RMSF and one kinetic parameter), separate intercepts for the two responses, and phylogenetic random effects on the intercepts. Furthermore, the models explicitly accounted for the uncertainty in both the temperature-corrected RMSF and the kinetic parameters. For each model, we executed three independent chains for a million generations, discarding the first 100,000 generations as burn-in, and sampling every 100 generations after that. After performing diagnostics for convergence and sufficient sampling of the posterior (as previously described), we estimated the correlations from the resulting variance-covariance matrices (Fig. S8).

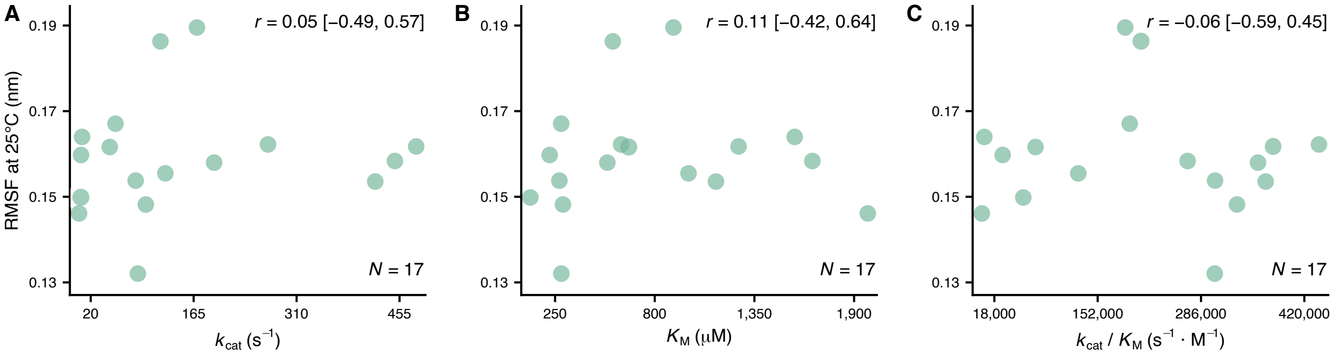

Figure S8: Temperature-normalised structural flexibility appears to be independent of kinetic parameters across 17 prokaryotic ADKs. The reported estimates stand for the mean of the posterior distribution of each correlation and its 95% HPD interval.

References

Branden, C. I., and J. Tooze. 2012. Introduction to protein structure. Garland Science.

Laskowski, R. A., M. W. MacArthur, D. S. Moss, and J. M. Thornton. 1993. PROCHECK: a program to check the stereochemical quality of protein structures. Journal of Applied Crystallography 26:283–291.

Muir, D. F., G. P. R. Asper, P. Notin, J. A. Posner, D. S. Marks, M. J. Keiser, and M. M. Pinney. 2025. Evolutionary-scale enzymology enables exploration of a rugged catalytic landscape. Science 388:eadu1058.

Rost, B. 1999. Twilight zone of protein sequence alignments. Protein Engineering 12:85–94.
